# Metabolic Adaptation of *Ralstonia solanacearum* during Plant Infection: A Methionine Biosynthesis Case Study

**DOI:** 10.1371/journal.pone.0036877

**Published:** 2012-05-16

**Authors:** Laure Plener, Pierre Boistard, Adriana González, Christian Boucher, Stéphane Genin

**Affiliations:** 1 INRA, UMR441 Laboratoire des Interactions Plantes-Microorganismes (LIPM), Castanet-Tolosan, France; 2 CNRS, UMR2594 Laboratoire des Interactions Plantes-Microorganismes (LIPM), Castanet-Tolosan, France; Universidad Pública de Navarra, Spain

## Abstract

MetE and MetH are two distinct enzymes that catalyze a similar biochemical reaction during the last step of methionine biosynthesis, MetH being a cobalamin-dependent enzyme whereas MetE activity is cobalamin-independent. In this work, we show that the last step of methionine synthesis in the plant pathogen *Ralstonia solanacearum* is under the transcriptional control of the master pathogenicity regulator HrpG. This control is exerted essentially on *metE* expression through the intermediate regulator MetR. Expression of *metE* is strongly and specifically induced in the presence of plant cells in a *hrpG*- and *metR*-dependent manner. *metE* and *metR* mutants are not auxotrophic for methionine and not affected for growth inside the plant but produce significantly reduced disease symptoms on tomato whereas disruption of *metH* has no impact on pathogenicity. The finding that the pathogen preferentially induces *metE* expression rather than *metH* in the presence of plant cells is indicative of a probable metabolic adaptation to physiological host conditions since this induction of *metE* occurs in an environment in which cobalamin, the required co-factor for MetH, is absent. It also shows that MetE and MetH are not functionally redundant and are deployed during specific stages of the bacteria lifecycle, the expression of *metE* and *metH* being controlled by multiple and distinct signals.

## Introduction

*Ralstonia solanacearum* is a Gram-negative soil-borne β-proteobacterium which is the causal agent of bacterial wilt, one of the most devastating bacterial plant diseases in the world. The strong impact of this pathogen results from its worldwide geographical distribution and its wide host range, since it affects more than 200 plant species, including important food crops such as tomato, potato, banana and eggplant [1]. *R. solanacearum* infects plants via wounds, the root tip, or cracks at the sites of lateral root emergence. The bacteria subsequently colonize the root cortex, invade the xylem vessels and reach the stem and aerial parts of the plant through the vascular system [2], [3]. *R. solanacearum* can rapidly multiply in the xylem up to very high cell densities, leading to wilting symptoms and plant death.

As soon as it enters the host root tissue, *R. solanacearum* has to face hostile environmental conditions due to plant defence reactions and to limited nutritional resources in the plant apoplasm. The pathogen relies on a complex regulation network that coordinates the expression of its various pathogenicity determinants according to the environmental stimuli [4]. A key player of this regulatory system is HrpG, which directly or indirectly controls the transcriptional induction of more than 350 genes in the presence of plant cells, including those directing the synthesis of the Type III Secretion System (T3SS) and effector substrates which is essential to pathogenesis [5], [6], [7]. HrpG integrates multiple environmental signals and controls, beyond the T3SS, many other bacterial functions that promote disease (reviewed in [8]).

In order to mobilize efficiently the host resources and to ensure its multiplication inside plant tissues, the bacterium has to deploy energy to set up the necessary elements required for the success of the infectious process. This implies that the pathogen has to be metabolically proficient, and little is known about the specific metabolic requirements once the pathogen invades its host. Some *R. solanacearum* mutants deficient for methionine, tryptophan or leucine [9], [10] or folate [11] biosynthesis were found to be avirulent on plants. However, because these mutants were auxotrophic, the loss of virulence resulted presumably from nutritional deficiency, which is the case if the host fails to provide an adequate source of the required metabolite in the infection court.

**Figure 1:**
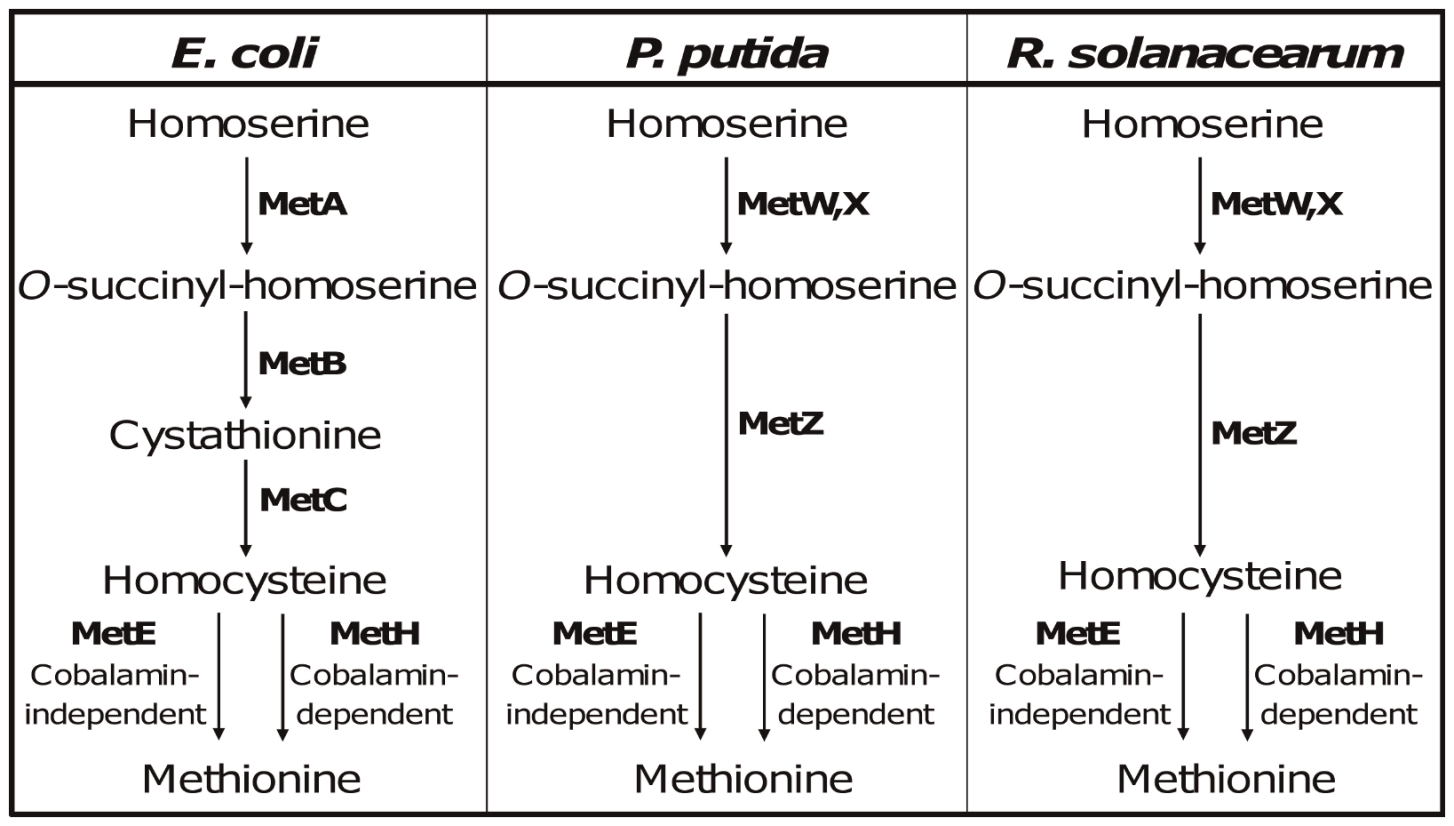
Pathways for methionine biosynthesis in *E. coli*, *P. putida* and *R. solanacearum*. The MetE and MetH methionine synthases catalyze the methylation of homocysteine to produce methionine, with methyltetrahydrofolate [5-methyl tetrahydropteroyltri-L-glutamate] being the methyl group donor [13].

Genetic robustness, which characterizes the constancy of the phenotype in face of heritable perturbations, has been attributed to two main causes. One is gene duplication and the other is the environmental specificity of some of the dispensable genes [12]. Since the host plant represents a specific environment for plant pathogens, we were interested to look for the possible existence of metabolic genes of *R. solanacearum* involved in the adaptation to the host plant. In this study, we studied the role of methionine biosynthesis in *R. solanacearum* pathogenicity, focusing our analysis on two enzymes, MetE and MetH, which perform a similar biochemical reaction and apparently look as functionally redundant. Methionine is a sulfur-containing amino acid which, apart from its function as a structural component of proteins plays also an important role in methyl transfer reactions via its derivative S-adenosylmethionine [13]. The methionine biosynthetic pathway and its regulation differ among various groups of organisms. Main differences are found in the nature of the acylhomoserine intermediate formed in the first step of the pathway and in the method of assimilation of the sulfur atom (Figure 1). From what can be deduced from the genomic data, *R. solanacearum* strain GMI1000 synthesises homocysteine from homoserine through the consecutive action of a homoserine-O-actetyltranseferase (encoded by *metX* and *metW*, RSc0027–RSc0026) and a homocysteine synthase (encoded by *metZ*, RSc1562). The first part of this pathway is different from the *E. coli* biosynthesis pathway and resembles to the one described in *P. aeruginosa* or *P. putida* [14]. The last step involves the methylation of homocysteine to produce methionine: this reaction is catalyzed by a cobalamin (vitamin B12)-dependent transmethylase, the *metH* gene product, or by a cobalamin-independent transmethylase, the *metE* (RSp0676) gene product. In GMI1000, MetH is encoded by two genes, *metHa* (RSc0295) and *metHb* (RSc0294), organized in an operon.

In *E. coli* (which does not synthesize vitamin B12), the availability of the vitamin cofactor determines which transmethylase is expressed since cobalamin represses the expression of *metE* [15]. Both the *metE* and *metH* genes, and *metA*, require an activator protein encoded by *metR* in order to be efficiently expressed [16]. Another regulatory protein, MetJ, acts as a transcriptional repressor of expression for all the genes of the methionine biosynthetic pathway [17]. *R. solanacearum* GMI1000 can synthesize cobalamin [18] and possesses a *metR* gene (RSp0677) lying just next to *metE* on the megaplasmid replicon, but does not harbour any gene related to *metJ*. Our data show that regulation of methionine biosynthesis is operated differently in *R. solanacearum* than in *E. coli*, and is controlled by the master pathogenicity regulator HrpG. We also present evidence that methionine biosynthesis significantly contributes to *R. solanacearum* pathogenicity, although methionine auxotrophy has limited or no impact on bacterial growth in plant tissues.

## Results

### HrpG Regulates the Expression of Methionine Synthase Genes

**Figure 2:**
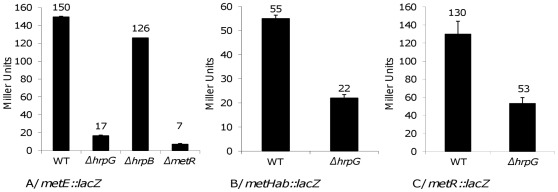
Expression of *metE*, *metHab* and *metR* in different genetic backgrounds. Expression of *metE* (A), *metHab* (B) and *metR* (C) was determined after 16 h of growth in minimal medium supplemented with glutamate at a 20 mM final concentration (A and B) and in the presence of plant cells (C). β-galactosidase activity is expressed in Miller units. Each measurement corresponds to the average of three replicates and bars indicate standard deviations.

The list of 112 genes positively regulated by HrpG in strain GMI1000, previously identified through transcriptomic analyses, comprised the methionine synthase cobalamin-independent gene *metE* [5]. To better investigate the role of HrpG in the regulation of methionine synthase genes, *lacZ* genomic fusions were created in the *metE* and *metHab* genes and expressed in different genetic backgrounds. The resulting strains were cultivated in minimal medium supplemented with glutamate which is known to induce HrpG activity [19]. Figure 2 shows the level of β-galactosidase activity monitored for each strain in this condition. The *metE* was more expressed in minimal medium than *metHab* genes since the β-galactosidase activity of the *metE::lacZ* fusion is three times superior to the one measured for the *metH::lacZ* fusion in the wild type strain. These data also confirmed that the expression of *metE* depends on the presence of HrpG but not on HrpB, the transcriptional activator of the T3SS, which is also under the control of HrpG (Figure 2A). The *metE::lacZ* fusion was expressed as much in the wild type strain as in the *hrpB* mutant background but expression was decreased by a 10-fold factor in the *hrpG* mutant. Expression of *metH* was also modulated by HrpG as the β-galactosidase activity of the *metH::lacZ* fusion was decreased by a 2.5-fold factor in the *hrpG* mutant compared to its expression in the wild type (Figure 2B). These results showed that HrpG controls expression of genes involved in the last step of methionine biosynthesis: this control is exerted independently from HrpB and appears tighter on *metE* than on *metHab*.

### HrpG-dependent Regulation of *metE* is Exerted via the Regulator MetR

In *E*. *coli*, expression of *metE* and *metH* genes is known to be controlled by the MetR regulator [16]. To investigate the role of MetR on *metE* expression, the *metE::lacZ* fusion was introduced in a *metR* mutant background and the β-galactosidase activity was monitored after growth of the strain in minimal medium supplemented with glutamate. Expression of *metE* was abolished in a *metR* mutant. These results confirmed that expression of *metE* in *R. solanacearum* is controlled by MetR as almost no β-galactosidase activity was measured in the *metR* mutant genetic background (Figure 2A). However, no significant decrease of *metHab* expression was measured in the *metR* mutant background (data not shown), showing that *metE* and *metHab* are differentially controlled by MetR.

Finally, a plasmid-borne *metR::lacZ* fusion was constructed and expression of this construct was reduced of a 2.5-fold factor in the *hrpG* mutant vs the wild-type strain, indicating that *metR* expression was dependent on HrpG (Figure 2C). Taken together these results suggested that the HrpG-dependent regulation of *metE* is indirect and mediated by MetR whereas the expression of *metHab* escapes this control. These results were also supported by the observation that the *metE* promoter possesses an *E. coli* MetR-like consensus recognition sequence (TGAANNNNNTTCA) [20] but no similar sequence was found in the *metH* gene promoter region.

### *R. solanacearum metE*, *metH* and *metR* Mutants are not Auxotrophic for Methionine

**Figure 3:**
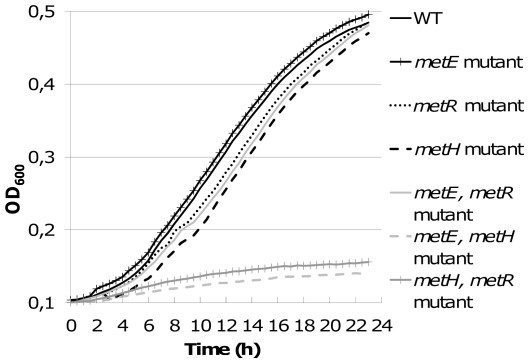
Growth curves of each single and double mutant in the *metE*, *metH* or *metR* genes. Growth was measured and compared to the wild type strain in minimal medium supplemented with glutamate at a 20 mM final concentration using a microplate reader. six replicates were measured for the non auxotrophic strains and three replicates were used for the auxotrophic strains. Results represent the mean value for all replicates at each time point. The *metE metH* and *metH metR* double mutant strains are auxotrophic for methionine.

As in other microorganisms possessing two methionine synthase genes, the *metE* and *metH* single mutants were not auxotrophic for methionine and showed similar growth rates as the wild type strain GMI1000 in minimal medium supplemented with glutamate (Figure 3). Here, the obvious functional redundancy of these genes explains the prototrophy phenotypes. A main difference between the MetE and MetH synthases is whether they require for activity cobalamin as a co-factor. We confirmed that the GMI1000 MetE and MetH synthases indeed behave as cobalamin-independent and –dependent enzymes, respectively. Disruptions of the *metE* and *metHab* genes were introduced in the cobalamin (*cobN*) mutant strain GMI 1783 (see Material & Methods). The resulting strains GMI1784 (*cobN metE*) and GMI1785 (*cobN metHab*) were cultivated on minimal medium. Only the cobalamin, *metH* double mutant was able to grow on minimal medium proving that the presence of *metE* gene enables the strain to synthesise methionine in the absence of cobalamin. Inversely, the cobalamin, *metE* double mutant was only able to grow when minimal medium was supplemented with cobalamin, proving that MetH requires the presence of cobalamin to synthesise methionine (Figure S1).

More surprisingly the *R. solanacearum metR* single mutant was not auxotrophic for methionine whereas the corresponding mutant is described to be auxotrophic in *E. coli* [16]. Indeed a *metR* mutant showed a growth rate in minimal medium similar to the *metE* and *metH* single mutants as well as to the wild type strain (Figure 3). To better understand the prototrophy of the *metR* mutant, double mutants were created and tested for their auxotrophy for methionine. As expected, the *metE metH* double mutant was auxotrophic for methionine, thus proving that no other enzyme is able to perform the last step of methionine biosynthesis pathway in *R. solanacearum*. On the other hand, the *metE metR* double mutant was not auxotrophic for methionine and showed no differential growth rate in minimal medium compared to the wild type strain. The prototrophy of *metE metR* double mutant compared to the auxotrophy of the *metE metH* double mutant suggested that in the *metE metR* double mutant the expression of *metH* genes was sufficient to relieve the auxotrophy for methionine in this strain. This hypothesis was validated by creating a *metR metH* double mutant, which was subsequently found to be auxotrophic for methionine (Figure 3). This result confirmed that the expression of *metH* in *R. solanacearum* is not dependent on MetR. Furthermore the observation that a *hrpG metH* double mutant is not auxotrophic for methionine suggested that in this mutant a residual expression of *metR* and, in turn, of *metE* is sufficient to relieve methionine auxotrophy.

### Expression of the Methionine Synthase Gene *metE*, but not *metH*, is Specifically Induced in the Presence of Plant Cells

**Figure 4:**
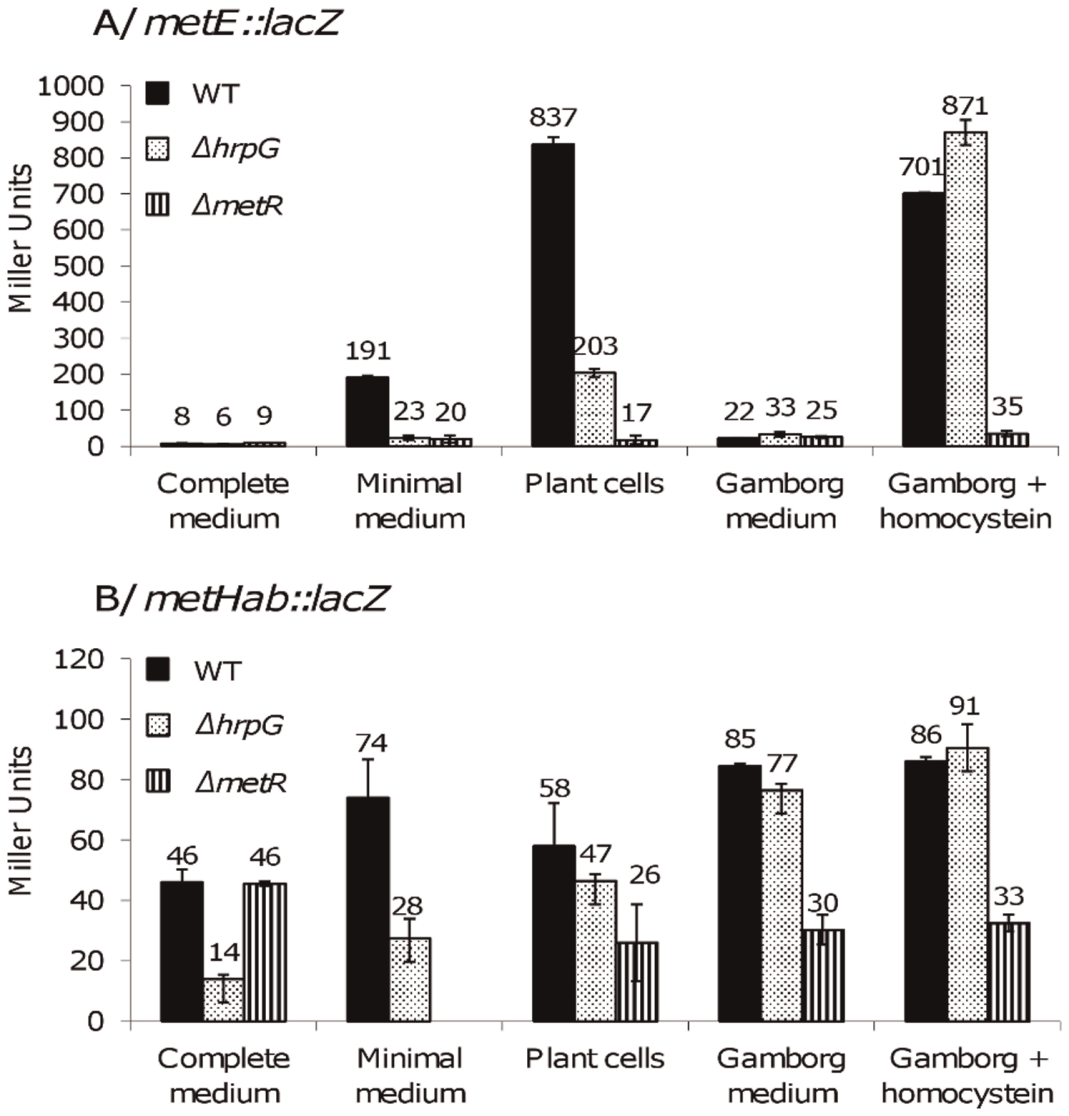
Expression of *metE* and *metHab* is differentially regulated in the presence of plant cells. Gene expression of *metE* (A) and *metHab* (B) was monitored after 16 h of growth in complete medium, minimal medium supplemented with glutamate at 20 mM final concentration, in Gamborg medium containing *Arabidopsis thaliana* cells, in Gamborg medium without plant cells and in Gamborg medium supplemented with homocysteine at 100 µM final concentration. β-galactosidase activity is expressed in Miller units. Each measurement corresponds to the average of three replicates and bars indicate standard deviations.

Since expression of *hrpG* is induced in the presence of plant cells [19], we determined whether the expression of methionine synthase genes was also specifically induced in this condition. Strains carrying reporter *lacZ* fusions in *metE* and *metH* genes were grown in the presence of *Arabidopsis thaliana* plant cells and in Gamborg medium, the synthetic medium used for plant cell growth. Expression level of the *metE* and *metHab* genes in these conditions was compared to their level of expression monitored in complete medium (which does not induce the expression of *hrpG*) and in minimal medium (which induces HrpG activity) [19]. The results showed that the expression of *metE* follows the HrpG induction pattern (Figure 4). The expression of the *metE::lacZ* fusion was strongly induced in the presence of plant cells compared to the expression in complete medium and this expression was decreased by a 4-fold factor in the *hrpG* mutant compared to the wild type strain. On the other hand, expression of *metHab* was not induced in the presence of plant cells compared to the non-inducing complete medium (Figure 4B). These data confirm that the two methionine synthase genes *metE* and *metH* have different expression patterns in *R. solanacearum* and that *metE* expression is specifically induced in the presence of plant cells, similarly to *hrpG* [19].

The reduced expression of *metE* in the *hrpG* mutant appeared less pronounced in the presence of plant cells than the one observed in minimal medium, and thus suggested that plant cell co-culture conditions could induce a residual *hrpG*-independent expression of *metE*. The finding that *metE* expression is completely abolished in presence of plant cells in a *metR* mutant (4-fold reduction in *hrpG* versus 50-fold in *metR*, see Figure 4A) also suggested that this *hrpG*-independent inducer was acting on *metR*.

### Homocysteine Induces the MetR-dependent Expression of *metE*

Since homocysteine is a co-activator of MetR regulatory protein in *E. coli* [21] we investigated whether homocysteine could also be an inducer of *metE* expression in *R. solanacearum*. Homocysteine was added to the Gamborg medium at a 100 µM final concentration and β-galactosidase activity was monitored for the different strains in this condition. Expression of *metE* was induced in the presence of homocysteine by a 30-fold factor in the wild type compared to the expression of *metE* in Gamborg medium without addition of homocysteine (Figure 4A). This induction was independent from HrpG as the expression of *metE* was similar in Gamborg medium supplemented with homocysteine in the wild type genetic background and in the *hrpG* mutant (Figure 4A). This induction of *metE* expression by homocysteine was only dependent on MetR as a 20-fold decrease in β-galactosidase activity was measured for *metE::lacZ* fusion between the wild type background and its *metR* mutant derivative. On the other hand, expression of *metHab* gene was independent from the presence of homocysteine as no induction of β-galactosidase activity is measured using the *metH::lacZ* fusion with or without homocysteine (Figure 4B).

### *metE* and *metR* Mutants are not Impaired for *in planta* Growth but have Reduced Pathogenicity

**Figure 5:**
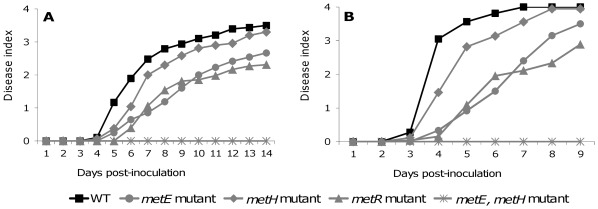
Disease progress curves of the various *R. solanacearum* methionine mutants on tomato plants. (A) For each strain 24 plants were inoculated using 50 mL of a bacterial suspension at 10^7^ CFU/mL for each plant. (B) For each strain 12 plants were inoculated by injection of 10^4^ CFU directly into the stem of the plant. Disease progress was rated using a disease index where 0 indicates healthy plants and 4 indicates a 100% wilted plant. For both tests 4 week old tomato plants were used. Results are representative of at least three independent experiments.

Because the methionine synthase genes belong to the HrpG virulence regulon, pathogenicity of the single disruption mutants for the *metE*, *metHab* and *metR* genes and the double mutant *metEH* was assayed on tomato and compared to the wild type strain. We first used an inoculation procedure by drenching the tomato plants with a bacterial suspension, which most closely reproduces the natural root infection process. In these conditions, the double mutant *metE metH* was completely avirulent and produced no disease symptoms (Figure 5A). This probably results from the inability of this mutant strain to invade or multiply within its host since no bacteria were recovered from the stem of inoculated plants (data not shown). The *metE* and *metR* single mutants exhibited a significantly reduced aggressiveness with symptom rates delayed from 2 to 3 days compared to the wild type strain (*p* = 0,007 for *metE* mutant and *p* = 0,002 for *metR* mutant). The *metH* mutant was not significantly altered in pathogenicity since the disease progress curve is almost similar to that of the wild-type GMI1000 (*p* = 0,574).

To investigate whether the survival in the soil and the root invasion steps were involved in the decreased aggressiveness of the different mutants, another mode of inoculation was used by injecting the bacteria directly into the stem of tomato plants. Using this mode of inoculation, the *metEmetH* double mutant remained completely avirulent and the *metE* and *metR* mutants exhibited a reduced aggressivity phenotype comparable to the one observed with the soil drenching inoculation method (Figure 5B) (*p*<0,001 for *metE* mutant and *p* = 0,002 for *metR* mutant). Again, no significant difference could be observed between the aggressiveness of the *metH* mutant strain compared to the wild type strain (*p* = 0,1). The pathogenic behaviour of these mutant strains was similar using both inoculation methods, thus suggesting that methionine biosynthesis is not critical in the first step of the infectious process but rather inside the plant, during the bacterial colonisation of host tissues.

**Figure 6:**
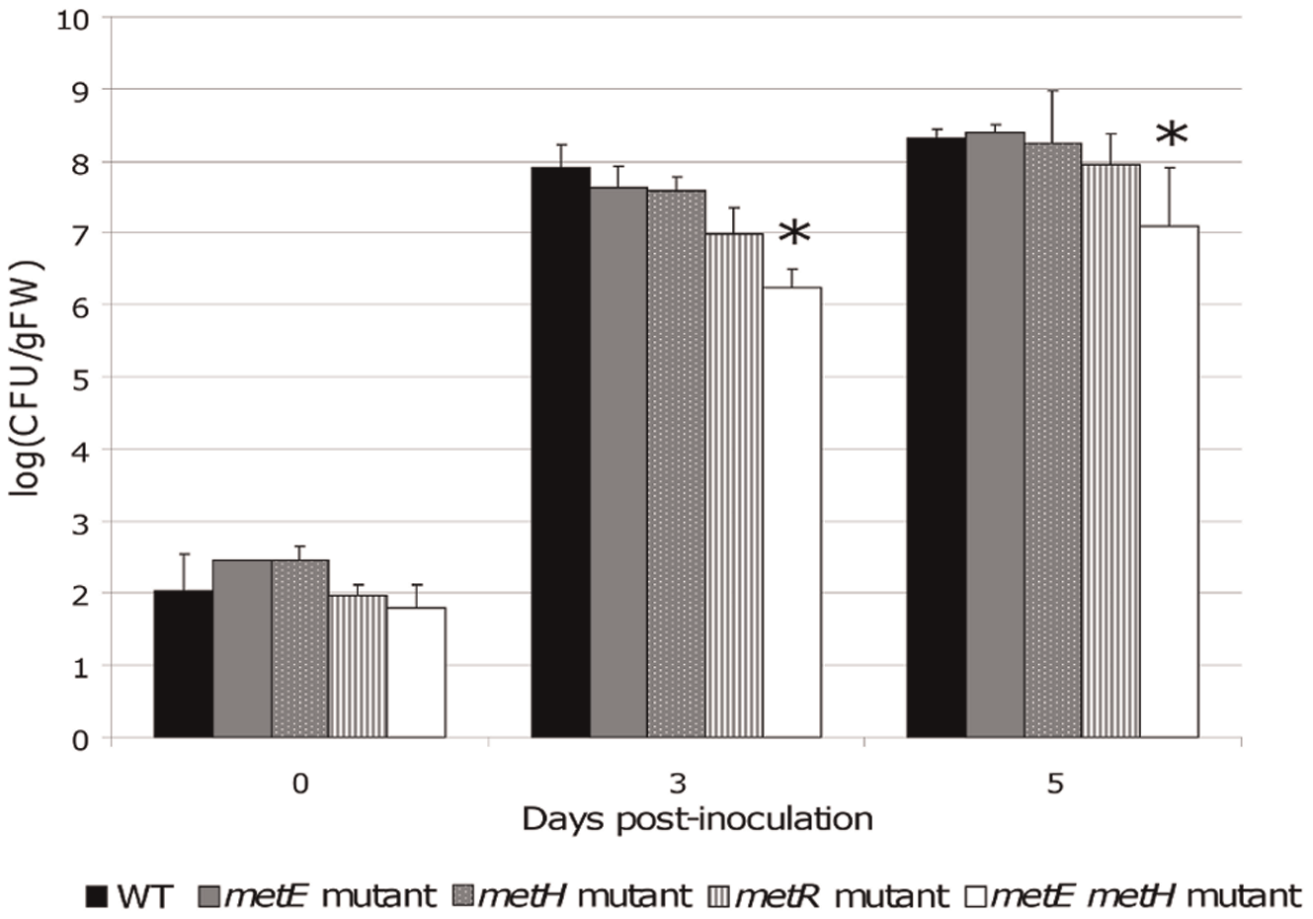
Determination of the bacterial growth in tomato plants of the different methionine mutants. For each strain 12 plants were inoculated by injection of 10^4^ CFU directly into the stem of the plant. 4 plants were used to estimate the bacterial density in the plant at 0, 3 and 5 days post-inoculation. Results are expressed in Log of colony number by gram of fresh matter log(cfu/gFW). Bars represent the standard deviation between the averages obtained for four plants from two independent experiments. Stars represent significant difference respectively to the wild type strain according to a Student test (*p-*value <0,05).

In order to evaluate whether methionine concentration could be a limiting factor in the plant stem preventing normal growth of the methionine mutants, the *in planta* growth of the different mutants was quantified. After inoculation directly into the tomato stems, the bacteria were recovered from the aerial part of the plants at 0, 3 and 5 days post inoculation (dpi) and serial dilutions were plated on BG agar medium for bacterial enumeration. Methionine auxotrophy of recovered bacteria was checked on synthetic medium deprived from methionine to ensure that no reversion in the *metEmetH* mutant occurred *in planta*. The results showed that the single mutants in *metE*, *metR* and *metH* genes have a similar growth to the wild type strain and reach a bacterial density of 10^8^ CFU gFW^−1^ at 5 dpi (Figure 6). More surprisingly, the *metEmetH* double mutant was also able to multiply in the plant stem. Methionine concentration in the tomato xylem sap was quantified around 3.7 µM [22] and we observed that a GMI1000 *metEmetH* mutant is still able to grow on a synthetic minimal medium supplemented with 3.7 µM methionine, albeit less efficiently than the wild-type strain (Figure S2). This probably explains why the bacterial population of this double mutant is statistically inferior to the one measured for the other strains at 3 and 5 dpi, but remains able to multiply inside the plant to reach a density of 10^7^ CFU/gFW at 5 dpi. These results indicated that the last step of methionine biosynthesis is necessary for the full pathogenicity of *R. solanacearum* and revealed that the reduced aggressiveness of the *metE* and *metR* prototrophic mutants did not result from impaired growth in the plant xylem.

## Discussion

### Part of the Methionine Biosynthesis Pathway is Under the Control of the Pathogenicity Regulon in *R. solanacearum*

The OmpR family regulatory protein HrpG plays a key role in coordinating different categories of pathogenicity genes required to overcome the plant defence responses and to survive in the intercellular spaces during the parasitic stage inside the plant [8]. HrpG was originally identified as a transcriptional regulator of the T3SS in *R. solanacearum* and further studies identified other regulatory targets more generally involved in the adaptation to life in the host [5]. This study uncovers a direct link between basal metabolism and pathogenicity in *R. solanacearum*, revealing that methionine metabolism is specifically activated once the bacteria are in the presence of plant cells and is genetically connected through the HrpG regulon to other essential pathogenicity determinants such as the T3SS. The observation that no other amino acid biosynthetic pathway than methionine appears to be controlled by HrpG from the transcriptomic data [5], indicates that HrpG does not stimulate a general activation of *R. solanacearum* metabolic pathways during infection but specifically the final step of the methionine biosynthesis pathway. Interestingly, a recent report on the transcriptomic characterization of the regulon controlled by the HrpG paralogue in *Xanthomonas axonopodis* pv. *citri* revealed that expression of *metE* is also *hrpG*-dependent in this plant pathogen [23], thus reinforcing the hypothesis of a specific requirement of a proficient methionine biosynthesis pathway in these bacteria during plant infection.

In this study, we provide evidence that HrpG controls the expression of the major methionine synthase gene during infection, *metE*, through another regulatory gene, *metR*. Trans-activation experiments in a heterologous *E. coli* system confirmed that *metR*, as *hrpB*, is a direct HrpG regulatory target (L.P. & S.G, unpublished results). *metR* is an ubiquitous regulator of the methionine biosynthetic pathway among Proteobacteria and this study provides the first example of the control of *metR* by a central pathogenicity regulator. Some of the regulatory mechanisms revealed in this study also differ from what is known in *E. coli* since in *R. solanacearum* the control exerted by the MetR regulator appears less essential to the biosynthesis: first, a GMI1000 *metR* mutant is not auxotrophic for methionine, contrary to what described for *E. coli* [16], and second, the genes involved in the first part of the biosynthesis pathway such as *metX* do not harbour any MetR recognition sequence in their promoter region, whereas the expression of this enzyme is controlled by MetR in *E. coli* [21], [24]. In *R. solanacearum*, MetR curiously seems to modulate only the expression of *metE* in the methionine biosynthesis pathway in response to a plant signal. Disruption of *metR* affects pathogenicity, but not more than what observed for a *metE* mutant, suggesting that the delayed pathogenicity phenotype of the *metR* mutant is only due to the control exerted on *metE*.

**Figure 7:**
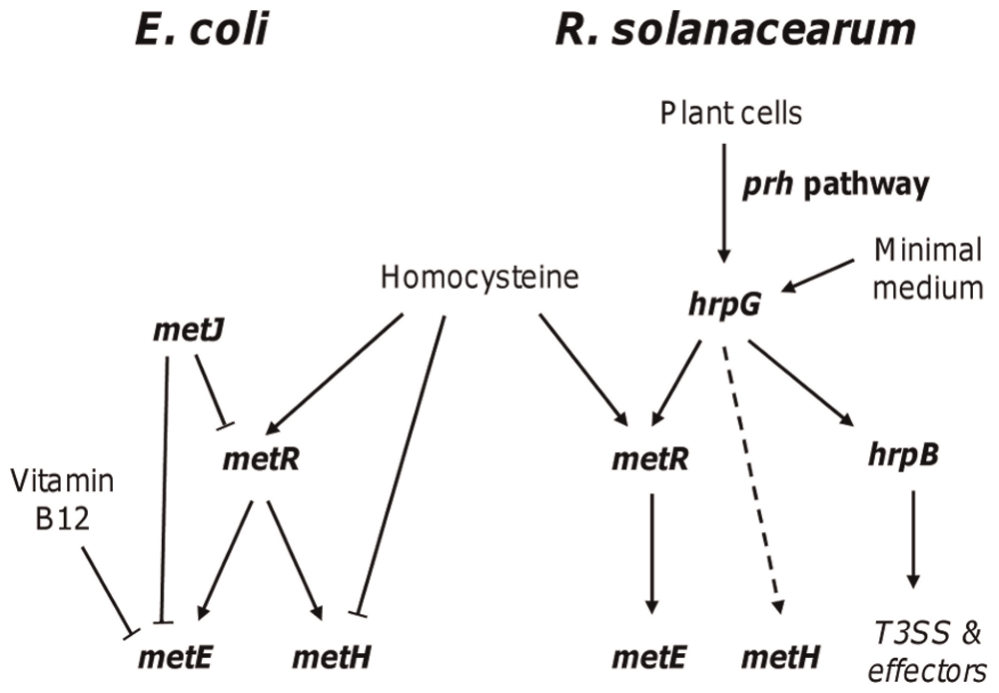
Comparison of the regulation of the methionine biosynthetic pathway in *E. coli* and *R. solanacearum*. Arrows symbolize positive regulation and broken lines negative regulation. The dashed arrow indicates that control of HrpG on *metH* is only partial.

MetR activity is enhanced by the metabolic inducer homocysteine, which results in increased expression of *metE*. Our data suggest that homocysteine is also present in plant cell co-culture conditions. Indeed, the expression of *metE* in the presence of plant cells is decreased by a 20-fold factor in the *metR* mutant compared to the *hrpG* deletion background (Figure 4), indicating that the MetR co-factor homocysteine induces a residual expression of *metE* in the *hrpG* mutant. On the other hand, expression of *metH* appears to be independent from homocysteine, in accordance to what is known in *E. coli*, where *metH* expression depends on MetR but it is not induced by the presence of homocysteine [25]. This difference for *metR* function in *R. solanacearum* and *E. coli* probably depends on the divergent environmental cue driving expression of methionine synthase genes (Figure 7). In *E. coli* a major constraint is to differentially control the expression of the two redundant enzymes depending on the availability of cobalamin in the milieu (since *E. coli* does not synthetize Vitamin B12) [26]. Conversely, *R. solanacearum* requires efficient methionine synthesis once inside the host environment, and *metR* appears to have been placed under *hrpG* control as a relay to specifically induce *metE* expression.

### A Metabolic Adaptation to Host Environmental Conditions

It is noteworthy that the control of HrpG leads to a very high expression of *metE*, the cobalamin-independent enzyme, rather than *metH*, the cobalamin-dependent methionine synthase. Gene expression data confirm that in the presence of plant cells expression of *metE* is strongly induced and results in ten-fold more expression than *metH* (Figure 4). Cobalamin is not synthetized by plants and only the cobalamin-independent methionine synthase activity has been described in higher plants [27]. It is therefore tempting to speculate that *R. solanacearum* mobilizes preferentially *metE* during the infection process to synthesize methionine in a cobalamin-deficient environment since the ability to synthesize cobalamin, one of the largest known non-polymeric natural products, requires a substantial energetic investment, necessitating the activity of ∼30 genes [28]. Accordingly, disruption of *metH* does not affect pathogenicity. The MetH enzyme was probably conserved through evolution because catalysis via cobalamin is mechanistically more advantageous, the *k*_cat_ value for MetH being 50–100 times higher than for MetE [13]. Although they encode enzymes performing an identical biochemical reaction, the *metE* and *metH* genes are not functionally redundant in *R. solanacearum* since they are differentially mobilized during the parasitic or saprophytic phases. This picture provides an example of a tight metabolic adaptation of the pathogen to specific conditions encountered in its host.

It is also possible that the strong expression of *metE* in the presence of plant cells counteracts part of the plant defense response. ROS (Reactive Oxygen Species) are important components of the host immune response [29], and many pathogens need to prevent and overcome oxidative stress in order to establish and maintain infections. The use of ROS as antimicrobial agents by the immune system is based on the high reactivity of this species with various cellular components, such as the MetE enzyme which is known to be very sensitive to ROS [30]. It is established that *R. solanacearum* encounters an oxidative environment during tomato infection [31] so it can be hypothesized that the specific *hrpG*-dependent activation of *metE* can be a mean for the pathogen to overcome the toxic affect of the plant oxidative burst. The finding that HrpG also activates the transcription of the *katE* catalase involved in the direct detoxification of ROS [5] supports this hypothesis.

### How Methionine Biosynthesis Contributes to Pathogenicity?

In addition to *R. solanacearum*, *met* auxotroph mutants have been described in other plant pathogens such as *Pseudomonas syringae* [32], [33] and *Agrobacterium tumefaciens* [34]. Whereas *A. tumefaciens* and *P. syringae* pv *phaseolicola met* mutants were avirulent, *P. syringae* pv *syringae met* genes were not tested for virulence but were shown to be required for optimum epiphytic fitness [35]. Compared to these former studies, a major difference is that the *metE* or *metH* single mutants are not methionine auxotrophs and our observations incite to think that the methionine biosynthetic pathway contributes to *R. solanacearum* aggressiveness not only by promoting bacterial growth. The strongest argument supporting this view is that at 5 dpi, the *metE* and *metR* mutants reach population levels similar to the wild-type parent, whereas significant differences in the rate of symptom development are already visible at this time point. This indicates that bacterial multiplication *in planta* and wilt symptom production can be dissociated in *metER* mutants in the experimental conditions used. Future investigations should determine if this behavior is variable among various host plants and whether bacterial multiplication of *metER* mutants is directly linked to the methionine concentration inside these hosts. Finally, methionine biosynthesis appears to be an essential requirement for pathogenicity of phylogenetically distant *R. solanacearum* strains differing in their host range spectrum: a recent study led on strain IPO1609, which carries a large genomic deletion comprising the *metER* genes, was shown to be hypovirulent on potato and this reduced virulence was partly dependent on *metER* genes [36].

The reason for a specific requirement of methionine biosynthesis in disease symptom production is currently not understood. We investigated whether T3SS-dependent secretion was altered in a *metE* mutant but no quantitative difference in the secretion of T3E Gala7 and PopP1 was observed after Western blot analysis (data not shown). Similarly, no significant difference was recorded after monitoring on test plates the activity of plant cell wall-degrading enzymes such as polygalacturonase or cellulase secreted through the general secretion pathway. Methionine is the precursor of SAM (*S*-adenosyl methionine) [13], the principal methyl group donor in cells, which is implicated in numerous cellular processes. It is therefore possible that reduced methionine biosynthesis impacts multiple cellular activities. One possible hypothesis to explain the role of MetE in the control of virulence is that SAM is a substrate of the PhcB methyltransferase which synthesizes 3-hydroxy palmitic acid methyl ester (3-OH PAME), the *R. solanacearum* virulence quorum sensing signal [37]. A reduced methionine synthase activity at a critical step of infection would limit the biosynthesis of 3-OH PAME and consequently delay the production of virulence factors such as exopolysaccharide. Another possibility relies on the fact that methionine is the direct precursor of polyamines such as spermidine and spermine, which are small polycationic compounds associated with a variety of biological processes. During the recent years, evidence has accumulated on the role of polyamines in microbial pathogenesis, survival in the host and oxidative stress tolerance [38], [39]. Finally, a third scenario is that during infection the MetE enzyme principally uses homocysteine and 5-methyl tetrahydropteroyltri-L-glutamate (methyltetrahydrofolate) of plant origin as substrates. This MetE-induced depletion of plant metabolites could alter the plant cell homeostasis and contribute to disease development. Future studies should address whether activation of the methionine pathway during plant infection by *R. solanacearum* is devoted to the synthesis of a specific factor or more generally contributes to bacterial fitness.

## Materials and Methods

### Bacterial Strains, Plasmids and Growth Conditions

Relevant characteristics of the plasmids and bacterial strains used in this work are listed in Table 1. *E. coli* strains were grown at 37°C in Luria Bertani medium [40]. *R. solanacearum* strains were grown in complete BG medium or in MP minimal medium supplemented with glutamate at 20 mM final concentration [41]. When needed, antibiotics were added to the media at the following final concentrations (mg/l): kanamycin (Km) 50 for *R. solanacearum*, spectinomycin (Sp) 40 for *R. solanacearum*, gentamycin (Gm) 10 for *R. solanacearum*, tetracyclin (Tc) 10 for *R. solanacearum*, ampicillin (Ap) 100 for *E. coli*.

**Table 1:** List of strains used in this study.

Strain or plasmid	Genotype	Reference or source
***E. coli***		
Top10	F- *mcrA Δ(mrr-hsdRMS-mcrBC) φ80lacZΔM15 ΔlacX74 nupG recA1 araD139 Δ(ara-leu)7697* *galE15 galK16 rpsL endA1 λ^−^,* Str^R^	Invitrogen
***R. solanacearum***		
GMI1000	Wild-type strain	[48]
GMI1525	*hrpB::Ω* mutant, Sp^R^	[49]
GMI1690	*metHab::lacZ,* Gm^R^	This work
GMI1754	*metE::lacZ,* Gm^R^	This work
GMI1755	GMI1000 *ΔhrpG* mutant	[5]
GMI1780	*metHab::Tn5 mutant*, Km^R^	This work
GMI1781	*metE::Ω* mutant, Sp^R^	This work
GMI1782	*metE::Ω*, *metHab::Tn5* mutant, Sp^R^ Km^R^	This work
GRS512	*metR::Ω* mutant, Sp^R^	This work
GRS543	*metR::Ω*, *metE::lacZ*, Sp^R^ Gm^R^	This work
GRS544	*metR::Ω, metHab::lacZ*, Sp^R^ Gm^R^	This work
GMI1783	*cobN::Tn5* mutant, Km^R^	This work
GMI1784	*cobN::Tn5, metE::Ω* mutant, Sp^R^ Km^R^	This work
GMI1785	*cobN::Tn5, metHab::lacZ* mutant, Gm^R^ Km^R^	This work
***Plasmids***		
pBluescript KS(+)	Cloning vector, Ap^R^	Stratagene
pGEM-T	Cloning vetcor, Ap^R^	Promega
pLAFR6	pLAFR1 with *trp* terminators, Tc^R^	[50]
pCZ205	pUC18-derived vector used for *lacZ* fusion, Ap^R^	[45]
pCZ367	pUC18-derived vector used for insertional mutagenesis, Ap^R^ Gm^R^	[44]
pLP45	pGEM-T with *Ω* insertion in *metE* gene, Ap^R^ Sp^R^	This work
pLP71	pBluescript KS(+) with *Ω* insertion in *metR* gene, Ap^R^ Sp^R^	This work
pLP198	pLAFR6 with *metR::lacZ* fusion, Tc^R^	This work

### DNA Manipulations and Genetic Constructs

Standard recombinant DNA techniques were performed as described previously [40]. Restriction enzymes, DNA ligase and other DNA enzymes were used according to the manufacturers’ recommendations.

### Creation of *metE*, *metR*, *metH* and *cobN* Disruption Mutants

*metE* and *metR* genes were disrupted by insertion of an Ω cassette [42] conferring resistance to spectinomycin. A 960 bp fragment upstream *metE* was cloned after PCR amplification using primers metE-Xba 5′-TCTAGAGGATCTCGAAGCGGAACAG-3′ and metE-HindUP 5′-AAGCTTGGTCATTTCCATGGCC-3′. A 980 bp fragment downstream of *metE* was cloned downstream of the first fragment using primers metE-HindDW 5′-AAGCTTCCGCGAGGGCCTGCCG -3′ and metE-Not 5′-GCGGCCGCCGGCGACATCCACG-3′. The Ω cassette was inserted between the two fragments using *Hin*dIII restriction site, resulting in plasmid pLP45. A 1.6 kb fragment of *metR* gene was cloned after PCR amplification using primers metR-F 5′- TTCGCGCACCTCGTCGAACA-3′ and metR-R 5′- GCGCCCCGCGTTCTGGAC-3′. The Ω cassette was inserted at position 225 of the gene using *Eco*RV restriction site resulting in plasmid pLP71. Both constructions were linearized by *Xba*I and introduced into *R. solanacearum* wild type and mutant strains by natural transformation. Double recombination events were selected using spectinomycin resistance and checked by PCR.

The *metH* and *cobN* (RSp0626) mutants were isolated from the *R. solanacearum* GMI1000 MutantsDb database. MutantsDb is a database of approx. 5000 mutants generated through random insertion of the transposon EZ-Tn5<KAN-2>™ from Epicentre Biotechnologies (Madison, WI). Localisation of each individual insertion on the genome was determined by sequencing of its flanking region using an adaptation of the TAIL-PCR protocol from Qian *et al.* [43] and automated BlastN comparison with the genome sequence. Correct insertion of the transposon integration in *metH* and *cobN* was confirmed by PCR and sequencing.

### Creation of *metE*, *metH and metR* Reporter Strains

The *metE::lacZ* and *metH::lacZ* fusion were created by using integration plasmid pCZ367 [44]. An internal fragment of the *metE* gene was PCR amplified by using primers MetE-5 (5′-AAGCTTATGAGCTCAAGGGCTGGCTG-3′) and MetE-3 (5′-TCTAGACTCCGAATAGCACATGTGCG-3′) and cloned as a 938-bp *Hin*dIII-*Xba*I fragment into pCZ367. An internal fragment of the *metHa* gene was PCR amplified by using primers MetH-5 (5′-AAGCTTCCTGGTGAACCTGGTCGG-3′) and MetH-3 (5′-ACCGGATCCAAGGCGTTC-3′). These plasmids were used to transform *R. solanacearum* GMI1000 and a single integrative event was selected by using pCZ367 gentamicin resistance. Recombinant clones were then checked by PCR.

*lacZ* reporter fusions with the *metE* and *metH* genes were introduced into the different genetic backgrounds (Δ*hrpG*, *hrpB:*: Ω, and *metR:*: Ω) by transformation with the genomic DNA of the donor strains. Recombinant strains were selected on media with adequate antibiotics, and the correct genomic insertion of the *lacZ* fusion was checked by PCR.

The *metR::lacZ* fusion was created by using pCZ205 [45]. A 1 kb fragment of *metR* promoter sequence was PCR amplified using primers metRp-Hind (5′-TAAAGCTTCGTCGACCACGCCGGC-3′) and metRp-Xba (5′- TATCTAGAGAGATGGCGGATTTCGAG-3′) and cloned into pCZ205 using *Hin*dIII and *Xba*I restriction enzymes to create a *metR::lacZ* fusion. The *metR::lacZ* fusion was then introduced in a pLAFR6 vector using Hind*III* and Acc65i restriction enzymes resulting in pLP198. The plasmid was introduced into the different reporter strains by electroporation (2.5 kV, 200 W, 25 mF, 0.2-cm cuvette gap).

### Determination of Growth Rates

The growth rates of strains GMI1000 and the derivative mutants were measured in cultures growing in MP minimal medium supplemented with glutamate at a 20 mM final concentration. Overnight cultures grown at 28°C with 200 rpm shaking were used to inoculate 200 µL of fresh medium with an initial OD_600_ at 0.1. Bacterial growth was performed in 96-well plates and monitored using the FLUOstar Omega microplate reader (BMG Labtech), measures of OD_600_ was performed every 30 minutes. Six biological replicates were used for the non auxotrophic strains: wild-type strain, *metE*, *metR* and *metH* single mutants, and for *metE, metR* double mutant; three biological replicates were used for auxotrophic mutants: *metE, metH* and *metR, metH* double mutants. For each time point, the mean of the replicates was calculated.

### Plant Cell Cultures and Bacteria-plant Cell Cocultures

The *A*. *thaliana* At-202 (accession Col-0) cell suspension lines were grown in Gamborg B5 (Flow Laboratories). For the bacteria-plant cell co-cultivation, 10 ml samples of *Arabidopsis* cell suspensions were inoculated with *R*. *solanacearum* [19]. After 16 h of incubation at 28°C, the co-culture was filtered and the bacterial cells were recovered for β-galactosidase assays.

### β-Galactosidase Assays

β-Galactosidase assays were performed as described previously [19]. β-Galactosidase activity was determined and expressed in Miller units [46].

### Plant Tests

Pathogenicity tests by soil inoculation were performed by watering 4-week-old tomato plants (*Lycopersicum esculentum cv. Marmande*) with 50 ml of a suspension containing 10^7^ CFU ml^−1^. Pathogenicity tests by stem injection were performed by injecting 20 µl of a 10^6^ CFU ml^−1^ bacterial suspension into the stems of 4-week-old plants. Disease development was scored daily by using a disease index scale ranging from 0 for no symptoms to 4 for completely wilted plants.

To evaluate bacterial multiplication in plants, tomato stems were removed, weighed, sterilized, cut and placed for 30 min at 25°C in sterile water. Bacteria were enumerated by plating serial dilutions onto BG medium.

### Statistical Analysis of the Data

For pathogenicity assays, within-group Kaplan-Meier survival estimates (the total number of plants surviving [i.e., with disease index scores below 2] out of the total number inoculated for each group) were computed for each time interval in order to build Kaplan-Meier survival curves for each group. The log rank test was used to perform between-group comparisons, testing the equivalence of the Kaplan-Meier survival curves for a pair of groups [47]. When *p*<0,05 the survival curves were considered as significantly different.

For bacterial multiplication in plants, comparison between the wild –type and each mutant strain was performed using a Student’s *t* test; when *p*<0,05 the bacterial multiplication was considered as significantly different between the two conditions studied.

## Supporting Information

Figure S1 MetE and MetH are cobalamin-independent and –dependent enzymes, respectively.
(TIF)

Figure S2 Growth of the wild-type strain GMI1000 and methionine synthase mutants on minimal medium plates supplemented with methionine 25 µM, 3.7 µM or in absence of methionine.
(TIF)
