# Supplementary figures and images for: Metabolic Adaptation of Ralstonia solanacearum during Plant Infection: A Methionine Biosynthesis Case Study
Source: PLoS One. 2012 May 16;7(5):e36877. doi: 10.1371/journal.pone.0036877 (PMC3353975; doi:10.1371/journal.pone.0036877)

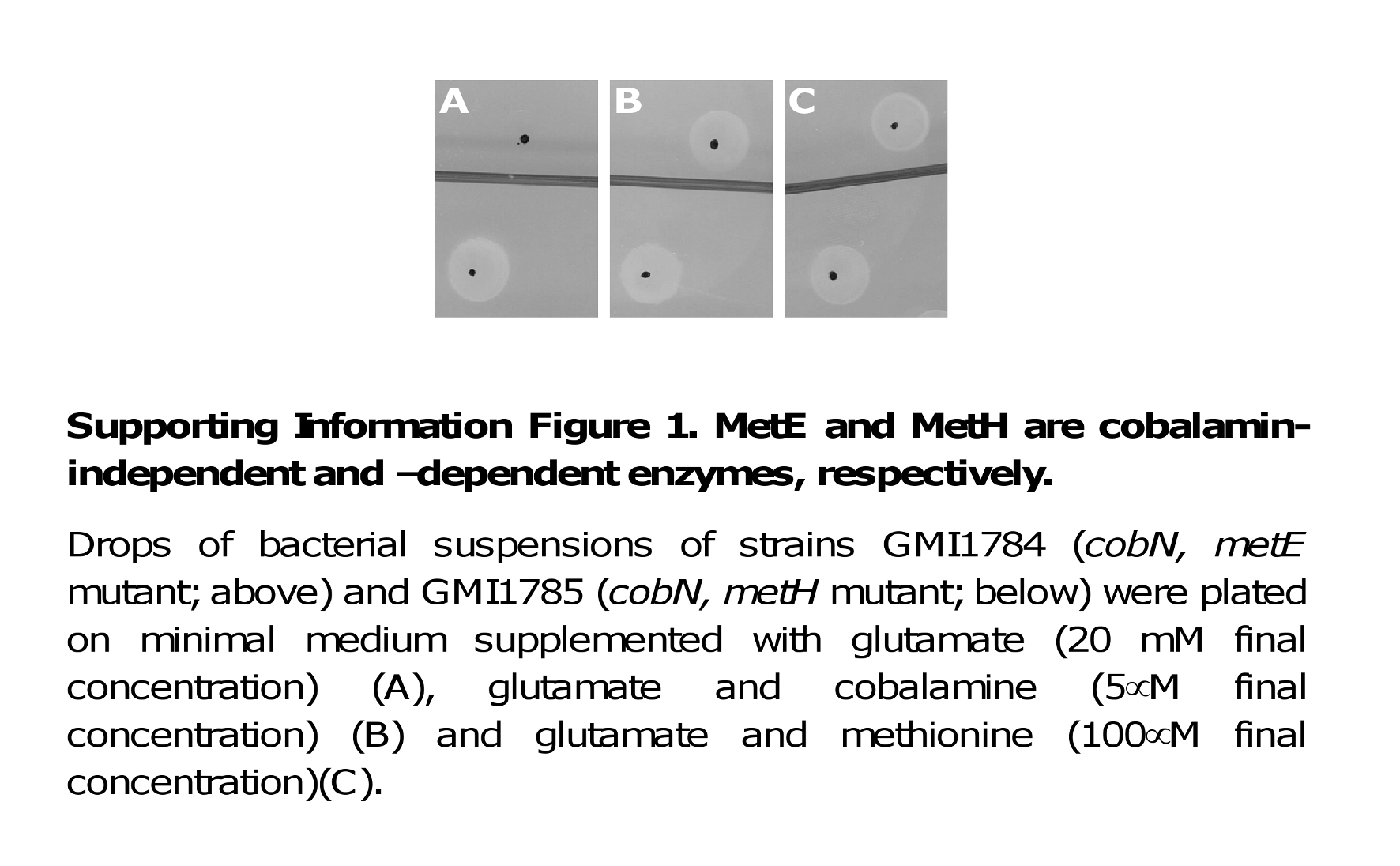

Supplement: Figure S1 — MetE and MetH are cobalamin-independent and –dependent enzymes, respectively. (TIF) [file pone.0036877.s001.tif]

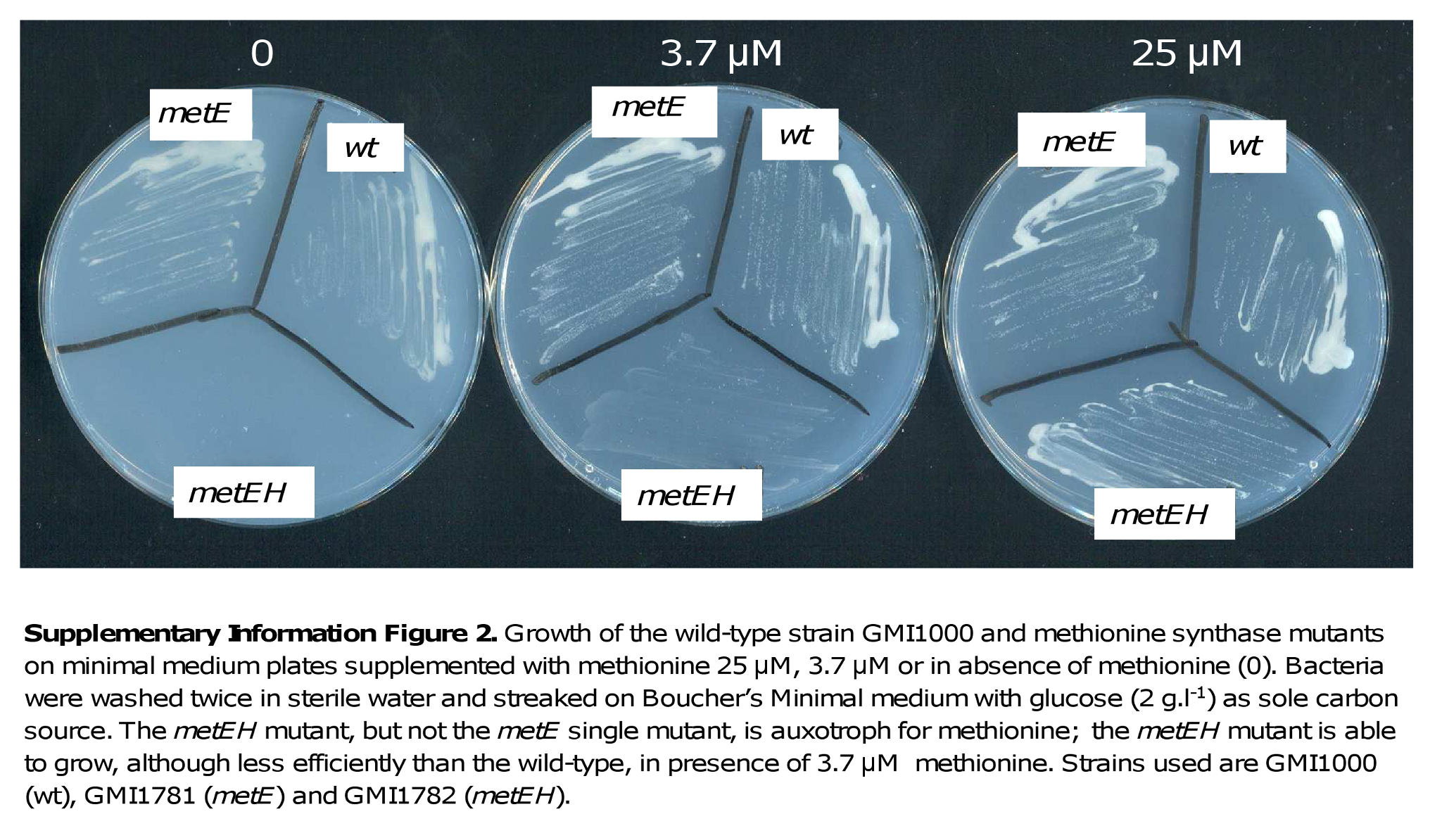

Supplement: Figure S2 — Growth of the wild-type strain GMI1000 and methionine synthase mutants on minimal medium plates supplemented with methionine 25 µM, 3.7 µM or in absence of methionine. (TIF) [file pone.0036877.s002.tif]
